# The Use of Technology-Based Simulation among Medical Students as a Global Innovative Solution for Training

**DOI:** 10.3390/brainsci14070627

**Published:** 2024-06-23

**Authors:** Francesco Guerrini, Luca Bertolino, Adrian Safa, Matilde Pittarello, Anna Parisi, Ludovica Vittoria Beretta, Elena Zambelli, Francesca Totis, Giovanni Campanaro, Lorenzo Pavia, Giannantonio Spena, Federico Nicolosi, Franco Servadei

**Affiliations:** 1 Unit of Neurosurgery, Department of Head & Neck Surgery, Fondazione Policlinico IRCCS San Matteo, 27100 Pavia, Italy; frague21@gmail.com (F.G.);; 2 Department of Biomedical Sciences, Humanitas University, 20090 Milan, Italy; luca.bertolino@st.hunimed.eu (L.B.); elena.zambelli@st.hunimed.eu (E.Z.);; 3 Department of Medicine and Surgery, University of Milano-Bicocca, 20900 Milan, Italy; 4 Department of Neurosurgery, IRCCS Humanitas Research Hospital, Rozzano, 20089 Milan, Italy

**Keywords:** education, neurosurgery, surgical training, technical skills, simulation

## Abstract

Background: Technological advancements have been rapidly integrated within the neurosurgical education track since it is a high-risk specialty with little margin for error. Indeed, simulation and virtual reality during training can improve surgical performance and technical skills. Our study aims to investigate the impact of neurosurgical technology-based simulation activities on medical students. Methods and Materials: The “Suturing Mission–The Symposium” was a three-day event held at Humanitas University. Participants had access to live-streamed conferences held by worldwide experts in several fields of neurosurgery and practical simulations of dura mater sutures, microvascular anastomosis, and augmented reality neurosurgical approaches. An anonymous survey was conducted at the beginning and end of the event. Results: 141 medical students with a mean age of 21 participated. After the course, 110 participants (77.5%) showed interest in pursuing a surgical path, with a great prevalence in those who had planned to have a surgical career before the event (88.7% vs. 41.4%, *p* < 0.001). Participants were also asked about their comfort levels while handling surgical instruments, and a good outcome was reached in 72.7% of participants, with a significant difference between those who had previously attended a suture course (87.8% vs. 66.3%, *p* = 0.012). Conclusion: Training sessions on surgical simulators were effective in increasing participants’ interest in pursuing a surgical path, improving their understanding of postgraduate orientation, and boosting their confidence with surgical instruments.

## 1. Introduction

Nowadays, neurosurgery does not represent a surgical specialty only, but also a fascinating and technologically growing field that it is often feared by medical students due to the traditional stereotypes of a technically complex, competitive field, with male preponderance [1], a high workload, risk of burnout [2], and susceptibility to litigation [3].

A possible, but difficult, solution could be increasing exposure to the neurosurgical environment before graduation [4,5,6]. Moreover, educational activities, interest-based groups, and collaborations are also suggested as a solution to ameliorate the perception and exposure of medical students to neurosurgery [4].

Technological advancements have been rapidly integrated with the neurosurgical field since it is a high-risk specialty with little margin for error. Indeed, it has been demonstrated that simulation and virtual reality during training can not only improve surgical performance and technical skills but also increase confidence in residents.

The integration of simulation could be useful in both the training and the performance of surgical procedures, allowing a faster learning curve, improving conceptual understanding of complex anatomy, and enhancing visuospatial skills for the developing neurosurgeon [5].

An ex vivo simulation study has been proven effective for improving students’ motivation to pursue a neurosurgical career [6]. However, there is a lack of published experiences with technology-based new simulation settings to expose medical students to some aspects of neurosurgery and contribute to shaping their perspectives and career choices. Introducing technology-based simulations early in medical school could bridge the gap between theory and practice in neurosurgery. This approach can help students make informed decisions about their career path, potentially reducing residency dropouts.

For this reason, we have conducted a study aiming to investigate the impact of neurosurgical technology-based simulation activity on medical students with no restriction of age or background, with respect to their careers and awareness of personal interests and manual abilities.

## 2. Materials and Methods

**Figure 1:**
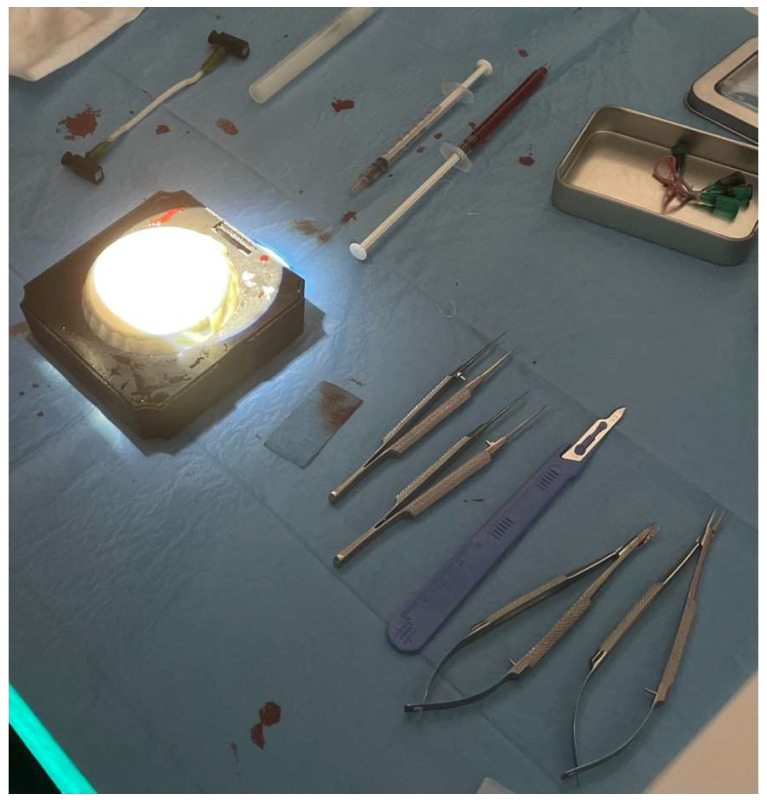
Microsuturing instruments used: micro-scissor, micro-forceps with teeth, micro-forceps without teeth, needle-holder, disposable scalpel, fake blood, and fake blood vessel.

**Figure 2:**
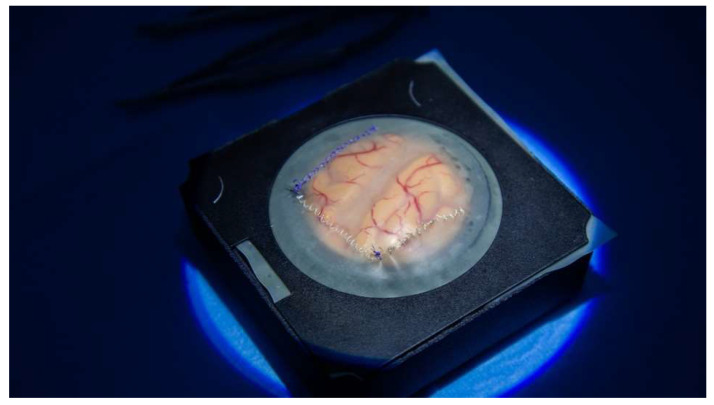
Simulator set for dura mater.

**Figure 3:**
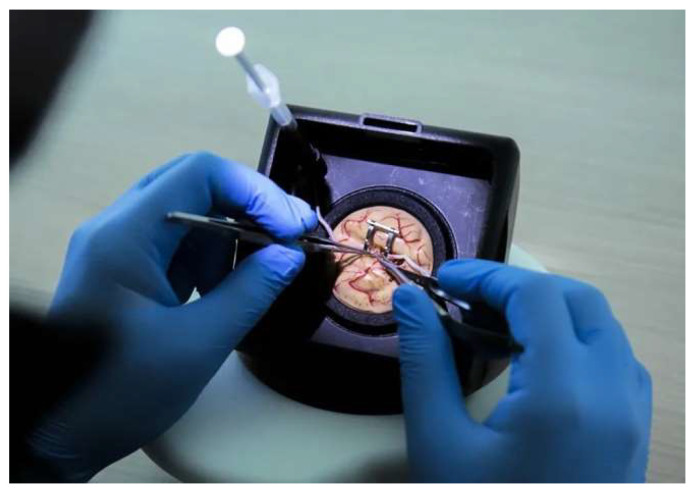
Simulator set for vessel anastomosis.

The “Suturing Mission–The Symposium” was a three-day event held at Humanitas University, Pieve Emanuele, Milan, Italy from the 11th to the 13th of November 2022, organized by a nonprofit network called Mission:Brain (https://www.missionbrain.org/about, accessed on 1 February 2023). Original data regarding students’ participation and survey results are reported in Supplementary Materials. The funds raised by the tickets required to participate are entirely devoted to sustaining similar events in other countries with limited facilities, where Mission:Brain associations are present. The symposium involves a series of both in-person and virtual conferences, internationally live-streamed, held by worldwide experts in several fields of neurosurgery and practical hands-on sessions of training on cadaver-free simulation technologies, developed and furnished via a free unlimited grant by UpSurgeOn, an Italian company based in Assago, Milan, Italy. Through voluntary recruitment, we have enrolled medical students from various academic years, from 1st-year students to 6th-year students. For the practical sessions, participants were all randomly divided into small groups and had the opportunity to rotate between two different stands where they could perform and practice dura mater sutures and microvascular anastomosis with microsuturing instruments (Figure 1). All the students performed the same task in the same way. In the dura mater suture session, they all had to perform a suture of a rectangular flap using a simple knot for two sides and a continuous knot for the third one, using a 4-0 suture thread (Figure 2). In the vessel anastomosis session, they all performed a side-to-side anastomosis via simple knot using an 8-0 suture thread (Figure 3). Mycro is the name of the simulator employed in this activity. It is a pocket-sized training kit for microsurgical techniques, which consists of a box with a high-fidelity representation of the brain, on which it is possible to integrate a membrane representing the dura mater (Figure 2) or a high-fidelity reproduction of a vessel (Figure 3). At a third station, they also had the opportunity to deepen their knowledge of neuroanatomy and neurosurgical approaches thanks to augmented reality and specific anatomical models developed by UpSurgeOn that integrate this technology. During each one of the practical sessions, both the suturing of the dura mater and the microvessel anastomosis, the participants were always under the guidance of trained staff and neurosurgery residents.

An anonymous survey was conducted at the beginning and the end of the event. It consisted of 28 questions, both open- and closed-ended. Six sections investigated anagraphical and background data, motivations, and medical and general skills held before and after the event. Missing data led to student exclusion from the final analysis (Table 1 and Table 2). Demographics and background data regarding the total number of participants, mean age, year of medical school, orientation, left- or right-handed, and vision problems were gathered. Data regarding any previous attendance of surgical intervention and suture courses were collected to assess the medical skills of the participants before the event. Participants’ general skills were assessed through a Likert-scale from 1 to 5 (1 = Strongly Disagree, 2 = Disagree, 3 = Neutral, 4 = Agree, and 5 = Strongly Agree) and concerned free-time activities sphere. After training on the simulators, participants were asked how much they agree with different statements, using the same scale from 1 to 5.

Statistical analyses were performed using R software v4.0.1. Chi-squared test (or Fisher’s exact test if applicable) was used to evaluate differences in proportions among subgroups of interest. Values of *p* < 0.05 were considered statistically significant. Data are presented as absolute and relative frequencies if not otherwise specified. A “bad outcome” reflected a score from 1 to 3, while 4 and 5 points were considered a “good outcome”.

## 3. Results

One hundred and forty-one medical students were included in the study. The mean age was 21 years (Table 3). The greater part of them were in their second (39.7%) and third (27.7%) year of medical school program (Table 3).

**Figure 4:**
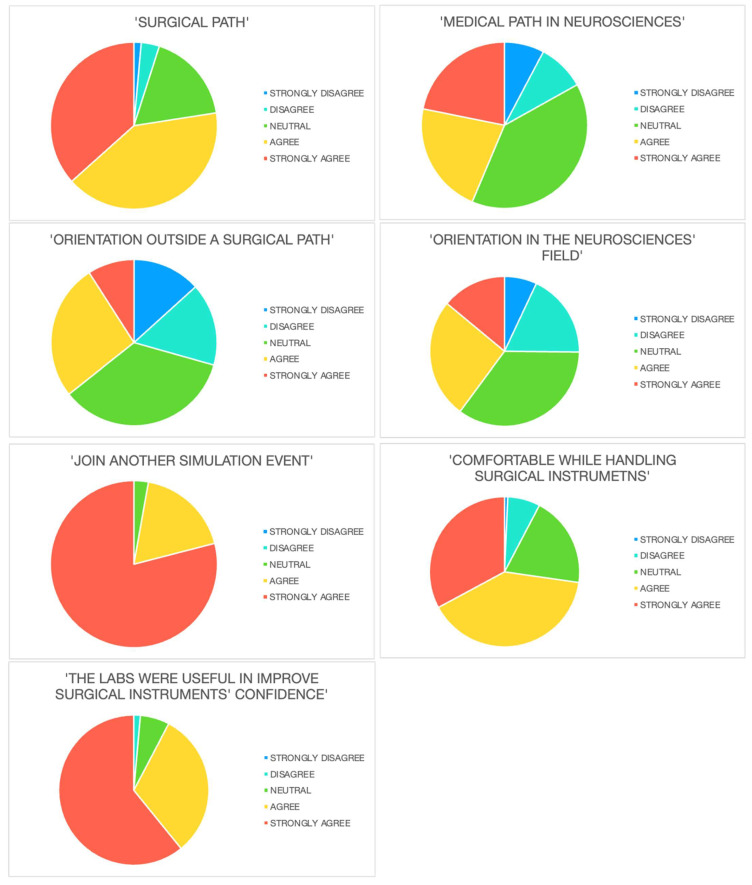
Pie charts representing post-simulation assessment of the participants.

**Figure 5:**
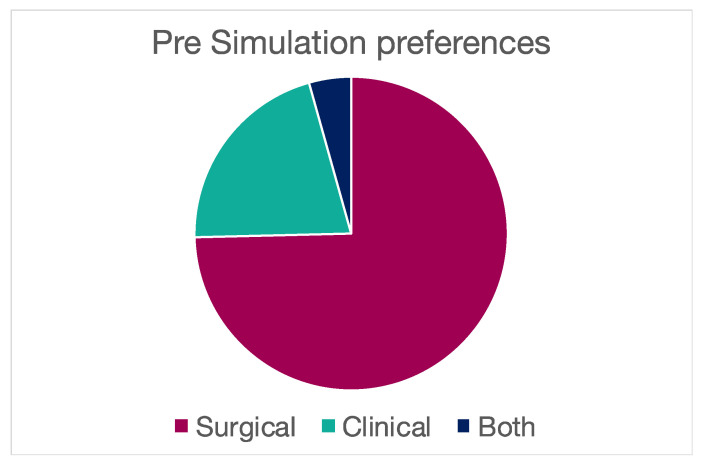
Specialty preference before simulation.

**Figure 6:**
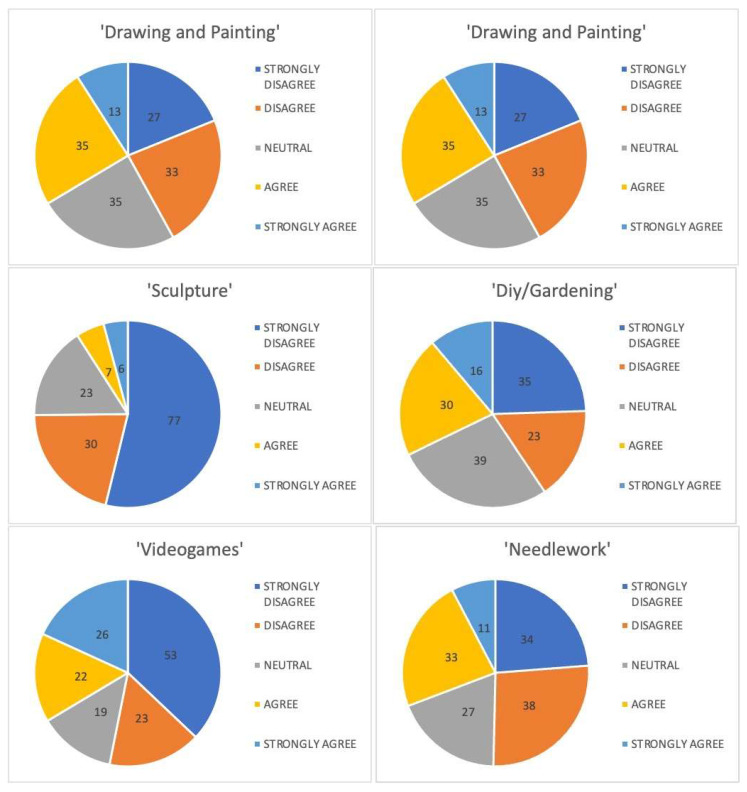
Pie charts representing general skills possessed by the participants before the event.

**Figure 7:**
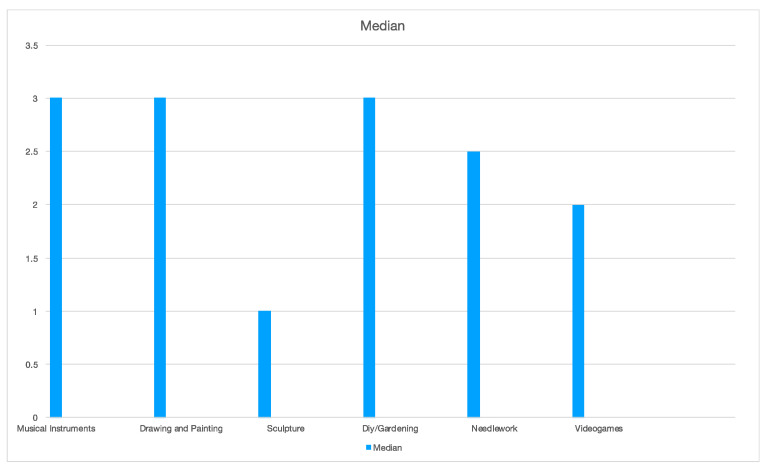
Median value of grades for each general skill assessed.

Before the simulation, specialty preferences were investigated among participants, showing 103 expressing an interest in pursuing a surgical specialty and 29 for a clinical specialty; a common interest in both surgical and clinical residency was present in 6 cases (Table 3) (Figure 4 and Figure 5). Around half of all participants showed a self-inclination for neurosciences, and 54% of them had already assisted in a surgical intervention. (Table 3) (Figure 6 and Figure 7) Myopia and hypermetropia were present in fifty-three and three cases, respectively. Forty-two students had a normal sight. As far as previous training is concerned, 41 students had already attended a suture technique course (Table 3, Figure 6).

After the course, 110 participants (77.5%) showed an interest in pursuing a surgical path, with a great prevalence in those who had planned to have a surgical career before the event (87.7% vs. 41.4%, *p* < 0.001) (Table 4, Figure 4).

The course reinforced the tendency to follow a medical neurosciences path in students who were interested in this field at the beginning (Table 4). Equally, it strengthened the intention to pursue other fields in the remaining participants (72.6% vs. 27.4%, *p* < 0.001) (Table 4). Moreover, pre-event students’ disposition influenced the tendency to follow a general neuroscience pathway (52.1% vs. 27.8%, *p* = 0.004) (Table 4).

When asked if the course was useful in orientating postgraduate pathways outside a surgical field, 92 participants (64.3%) gave a negative answer (Table 5, Figure 4). However, the pre-course specialty orientation of the students did not influence this issue (*p* = 0.259) (Table 4).

Participants were also asked about their comfort levels while handling surgical instruments. A good outcome was reached in 72.7% of participants (Table 5, Figure 4), with a significant difference between those who had attended a suturing course previously (87.8% vs. 66.3%, *p* = 0.012) (Table 4).

Finally, 97.2% of students agreed or strongly agreed to attend another simulation course (Table 5, Figure 4).

Participants were also asked about their general skills possessed before the event. Results are reported in Table 6.

## 4. Discussion

Simulation has always played a central role in human history in order to ameliorate abilities and reduce errors. We have traces dating back to 500 BC reporting simulation games to train decision-making and operational strategies and plan military tactics [7].

Simulation has been employed in many different fields, including aviation, the military, and medicine.

The first successful use of simulation in aviation began in the late 1920s with the Link Trainer, developed by Edwin Link, which allowed pilots to practice their skills in flying “blind” or in instrument training. Standards were implemented in aviation to create reliability in training and evaluation, making it possible to move to the use of predominately simulator-based training methods [8].

It is therefore straightforward to understand how, in a highly demanding, high-risk, technically difficult specialty, such as neurosurgery, the role of simulation plays a crucial role.

Simulation can be aimed at general neurosurgery as well as at specific neurosurgical sub-specialties, such as vascular neurosurgery, minimally invasive neurosurgery, brain tumor resection, pediatric neurosurgery, stereotactic radiosurgery, skull base neurosurgery, spine surgery, and functional neurosurgery [9].

Simulation techniques vary and span from physical models and visual reality, to mixed reality [10] and augmented reality. In the past, physical reality models included both animal and human cadaver models, playing a central role in the training of neurosurgeons but presenting several limitations such as safety risks, ethical regulations in material repairability, and failure to properly represent parameters of alive tissues [11].

Virtual reality models create a virtual world reproduced by recreating sounds and sensory stimuli experienced by the subject, which can be immersive or non-immersive [12]. It can be applied and aimed at multiple purposes in neurosurgery: neuronavigation [13], as a diagnostic tool [14], in neurosurgical training [15], for pain management [16], in rehabilitation [17], and in robotic neurosurgery [18]. The challenges of vital reality in neurosurgery are commonly linked to the technical complexity, applicability, adherence to real-life scenarios, ethics, and costs [19].

Mixed reality models combine a physical and a virtual component, making it possible for the user to interact with digital objects starting from real ones.

Augmented reality represents the physical world, and digital inputs are superimposed on it via a camera [20]. It is less immersive and more accessible than mixed reality, and in neurosurgery, it plays a crucial role in education, surgical planning, and neuronavigation and is growing in importance in spinal surgery [21].

Extend reality simulation tools show wide and heterogeneous applicability in neurosurgery and present a potential tool to seal the gap in neurosurgical training in low-income countries [19].

Given the wide variety of techniques and levels of difficulty provided, simulation systems can be used at various levels of expertise in the training of neurosurgery professionals. Simulation plays a valuable role in the training of residents [22] and in the planning and sub-specialization of neurosurgeons [23].

The role of neurosurgery simulation for medical students has previously been shown to have an educational benefit and impact on student motivation using an ex vivo pig model [6] or reusable microsurgery kits [24].

In this course, we used a peculiar simulation model, in which dura mater and brain reproductions are highly accurate and detailed. We think that a simulator with these attitudes could improve motor acquisition and automate psychomotor skills, in a cheap and risk-free framework [25].

Our study collects opinions from a large cohort of 141 medical students, with no limitations on age, previous experiences, or background. Firstly, the course resulted in being beneficial in helping students orient toward a surgical pathway, above all, in those who had expressed a surgical attitude at the beginning; nevertheless, when they were asked if the event reinforced a postgraduate inclination outside a surgical pathway, 64.3% of participants disagreed with this statement. This data should be read with the fact that 91% of trainees reported increasing confidence in handling surgical instruments, without a difference based on initial orientation. This is a very important finding, as it translates with the fact that such an organized course could, with the help of tutors like trainees or staff neurosurgeons, guide medical students to a postgraduate surgical world. In support of this, there was generalized enthusiasm, with around 80% of participants reporting being interested in taking part in future similar events. Obviously, those who had already attended a suture course felt more comfortable with surgical instruments, as they started at a higher level.

This course was centered on neurosciences, and it emerged from the fact that although only 43.7% of participants agreed or strongly agreed to pursue a postgraduate medical path in neurosciences, a significant difference was found according to pre-course attitudes. In fact, it reinforced the intention of those students who were already interested in this field.

These results are not only encouraging but propose our study as a model to introduce medical students to the surgery and neuroscience worlds. We know that in many medical schools, neurosurgical teaching is limited (12 h altogether in Italy and many other European countries); in some African countries, neurosurgery is completely out of the medical teaching curriculum. Hence, how can we increase the number of neurosurgeons in countries in need if medical students never cross this specialty? [26,27]. The event combined neuroscience conferences and practical simulations, which proved to be an effective way to introduce students to both theoretical and practical aspects of neurosurgery. In fact, early and aware carrier decisions could prove to be beneficial both to students, reinforcing their intention to follow a career in neurological sciences and to bypass stereotypes generally associated with surgery, and to neurosurgery residency programs since an early and more pondered carrier choice could limit the dropout rate from programs. Moreover, this particular training model, due to its low costs, has proven to be a valid training tool for young neurosurgery residents in low–middle-income countries to overcome their limited possibilities [28].

## 5. Limitations

The main limitations of our study concern the low number of participants and the lack of an objective tool to study the efficacy of the simulator technologies among medical students. Other limitations could be the absence of a control group, due to the setting of our event, and the absence of different staging sessions to better assess the performance of the participants. Moreover, there are potential cofactors, such as individual interests, experiences, educational environment, and background, that may influence the results. In this study we have only evaluated the following: having an interest to pursue a specialty career in a surgical or clinical field before the event, having a pre-event interest in pursuing a clinical career concerning the neurosciences, and whether or not they have participated in a suturing course before the event.

## 6. Conclusions

Overall, the result of this study shows that the introduction of medical students to the simulation training under the supervision of residents was effective in increasing students’ interest in pursuing a surgical and neuroscience path, improving their understanding of postgraduate orientation, and offering an alternative to a significant inclusion in the neurosurgical teaching at medical schools. However, long-term studies should be conducted to evaluate how these outcomes of interest will change and allow the addition of control groups to the simulation-trained cohort.

## Figures and Tables

**Table 1:** Questions related to personal information and background information.

QUESTION	ANSWERED REQUIRED
**PERSONAL INFO**	
Age	Open (numerical)
Nationality	Open
**STUDIES AND BACKGROUND**	
University	Open
Current year of studies	Open (numerical)
High school attended (type)	Open
Interest in pursuing a medical career	Clinical/surgical
If clinical, does it involve neurosciences?	Yes/No
If surgical, which field	Open
Dominant hand	Left/Right
Visual defects	Myopia/Hypermetropia/Astigmatism/None
**MOTIVATION**	
Why have you chosen to take part in this project?	Multiple choice

**Table 2:** Questions related to the skills possessed by the participants.

QUESTION	ANSWERED REQUIRED
**MEDICAL SKILLS POSSESSED BEFORE THE EVENT**	
Seen a surgical operation	Yes/No
Attended a suture technique course	Yes/No
**GENERAL SKILLS POSSESED BEFORE THE EVENT**	
I know how to play a musical instrument.	Likert scale from 1 to 5
I know how to draw or paint	Likert scale from 1 to 5
I know how to make a sculpture	Likert scale from 1 to 5
I am practical with DIY/gardening	Likert scale from 1 to 5
I am practical with needlework	Likert scale from 1 to 5
I play videogames	Likert scale from 1 to 5
If you play videogames (answer ≥ 3):	
Which kind of videogames?	Open
Have you ever used kinematic sensors?	Yes/No
**POST SIMULATION ASSESMENT**	
The event made me realize that I am interested in pursuing a surgical path.	Likert scale from 1 to 5
The event made me realize that I am interested in pursuing a medical path in the neurosciences.	Likert scale from 1 to 5
The event was useful to understand my postgraduate orientation outside of a surgical path.	Likert scale from 1 to 5
The event was useful to understand my postgraduate orientation in the neurosciences’ field.	Likert scale from 1 to 5
I would like to join another simulation event.	Likert scale from 1 to 5
I felt comfortable while handling the surgical instruments.	Likert scale from 1 to 5

**Table 3:** Demographics and specific characteristic of participants.

**Number of Participants**		141
**Mean Age**		21
**Year of Medical School**	1st	10
	2nd	56
	3rd	39
	4th	22
	5th	7
	6th	7
**Orientation**	Surgical	98
	Clinical	21
	Both	6
**Hand**	R	127
	L	15
**Vision**	M	53
	M/A	22
	H	3
	NONE	42
	M/A/H	1
**Surgical Intervention**	Yes	79
	No	67
**Suture Course**	Yes	41
	No	105
**Kinetic Sensors**	Yes	19
	No	49

**Table 4:** Data regarding the post-simulation assessment organized by pre-course attitude.

***The event made me realize that I am interest in pursuing a surgical path***			
*Precourse Attitude*	*Bad outcome (%)*	*Good outcome (%)*	*p value*
Surgical	13 (12.3)	93 (87.8)	***<0.001***
Clinical	17 (58.6)	12 (41.4)	
Both	2 (33.3)	4 (66.6)	
***The event made me realize that I am interest in pursuing a medical path in the neurosciences***			
*Precourse Attitude*	*Bad outcome (%)*	*Good outcome (%)*	*p value*
Neuro	26 (32.5)	45 (72.6)	***<0.001***
No neuro	54 (67.5)	17 (27.4)	
***The event was useful in understanding my postgraduate orientation outside of a surgical path***			
*Precourse Attitude*	*Bad outcome (%)*	*Good outcome (%)*	*p value*
Surgical	72 (67.9)	34 (32.0)	***0.259***
Clinical	16 (53.3)	14 (46.7)	
Both	3 (50)	3 (50)	
***The event was useful in understanding my postgraduate orientation in the neuroscience’s field***			
*Precourse Attitude*	*Bad outcome (%)*	*Good outcome (%)*	*p value*
Neuro	34 (47.9)	37 (52.1)	***0.004***
No neuro	52 (72.2)	20 (27.8)	
***I would like to join another simulation event***			
*Precourse Attitude*	*Bad outcome (%)*	*Good outcome (%)*	*p value*
Sutures	3 (7.3)	38 (92.6)	***0.072***
No sutures	1 (0.9)	100 (99.0)	
***I felt comfortable while handling the surgical instruments***			
*Precourse Attitude*	*Bad outcome (%)*	*Good outcome (%)*	*p value*
Sutures YES	5 (12.2)	36 (87.8)	***0.012***
Sutures NO	34 (33.7)	67 (66.3)	
***I felt comfortable while handling the surgical instruments***			
*Precourse Attitude*	*Bad outcome (%)*	*Good outcome (%)*	*p value*
Surgical	26 (24.5)	80 (75.5)	***0.204***
Clinical	12 (40.0)	18 (60.0)	
Both	1 (16.7)	5 (83.3)	

**Table 5:** Data regarding the post-simulation assessment.

**The event made me realize that I am interested in pursuing a surgical path**			
*Grade*	*Outcome (%)*	*Grade*	*Outcome (%)*
1	2 (1.4)	Bad	32 (22.5)
2	5 (3.5)		
3	25 (17.6)		
4	58 (40.8)	Good	110 (77.5)
5	52 (36.6)		
**The event made me realize that I am interested in pursuing a medical path in neuroscience**			
*Grade*	*Outcome (%)*	*Grade*	*Outcome (%)*
1	11 (7.8)	Bad	80 (56.3)
2	13 (9.2)		
3	56 (39.4)		
4	31 (21.8)	Good	62 (43.7)
5	31 (21.8)		
**The event was useful in understanding my postgraduate orientation outside of a surgical path.**			
*Grade*	*Outcome (%)*	*Grade*	*Outcome (%)*
1	19 (13.3)	Bad	92 (64.3)
2	23 (16.1)		
3	50 (35.0)		
4	38 (26.6)	Good	51 (35.7)
5	13 (9.1)		
**the event was useful in understanding my postgraduate orientation in the neuroscience’s field**			
*Grade*	*Outcome (%)*	*Grade*	*Outcome (%)*
1	10 (7.0)	Bad	86 (60.1)
2	26 (18.2)		
3	50 (35.0)		
4	37 (25.9)	Good	57 (39.9)
5	20 (14.0)		
**I would like to join another simulation event**			
*Grade*	*Outcome (%)*	*Grade*	*Outcome (%)*
1	0 (0.0)	Bad	4 (2.8)
2	0 (0.0)		
3	4 (2.8)		
4	26 (18.2)	Good	139 (97.2)
5	113 (79.0)		
**I felt comfortable while handling the surgical instruments**			
*Grade*	*Outcome (%)*	*Grade*	*Outcome (%)*
1	1 (0.6)	Bad	39 (27.3)
2	10 (7.0)		
3	28 (19.6)		
4	57 (39.9)	Good	104 (72.7)
5	47 (32.9)		

**Table 6:** Data on general skills possessed by the participants before the event.

**Musical Instruments**			
*Grade*	*N. of Participants*	*%*	*Median*
1	26	18.2	3
2	25	17.5	
3	22	15.4	
4	41	28.7	
5	29	20.3	
**Drawing and Painting**			
*Grade*	*N. of Participants*	*%*	*Median*
1	27	18.9	3
2	33	23.1	
3	35	24.5	
4	35	24.5	
5	13	9.1	
**Sculpture**			
*Grade*	*N. of Participants*	*%*	*Median*
1	77	53.8	1
2	30	21.0	
3	23	16.1	
4	7	4.9	
5	6	4.2	
**Diy/Gardening**			
*Grade*	*N. of Participants*	*%*	*Median*
1	35	24.5	3
2	23	16.1	
3	39	27.3	
4	30	21.0	
5	16	11.2	
**Needlework**			
*Grade*	*N. of Participants*	*%*	*Median*
1	34	23.8	2.5
2	38	26.6	
3	27	18.9	
4	33	23.1	
5	11	7.7	
**Videogames**			
*Grade*	*N. of Participants*	*%*	*Median*
1	53	37.1	2
2	23	16.1	
3	19	13.3	
4	22	15.4	
5	26	18.2	

## Data Availability

The original contributions presented in the study are included in the article/Supplementary Materials; further inquiries can be directed to the corresponding author.

## Supplementary Materials

The following supporting information can be downloaded at: https://www.mdpi.com/article/10.3390/brainsci14070627/s1.
